# Calmangafodipir for Prevention of Oxaliplatin-Induced Peripheral Neuropathy: Two Placebo-Controlled, Randomized Phase 3 Studies (POLAR-A/POLAR-M)

**DOI:** 10.1093/jncics/pkac075

**Published:** 2022-10-29

**Authors:** Per Pfeiffer, Maryam Lustberg, Jacques Näsström, Stefan Carlsson, Anders Persson, Fumiko Nagahama, Guido Cavaletti, Bengt Glimelius, Kei Muro

**Affiliations:** Department of Oncology, Odense University Hospital, Odense, Denmark; Smilow Cancer Hospital and Yale Cancer Center, Yale Medicine, New Haven, CT, USA; Egetis Therapeutics AB, Stockholm, Sweden; Egetis Therapeutics AB, Stockholm, Sweden; Egetis Therapeutics AB, Stockholm, Sweden; Solasia Pharma K.K., Tokyo, Japan; Experimental Neurology Unit, School of Medicine and Surgery, University of Milano-Bicocca, Monza, Italy; Department of Immunology, Genetics and Pathology, Uppsala University, Uppsala, Sweden; Department of Clinical Oncology, Aichi Cancer Center Hospital, Nagoya, Japan

## Abstract

**Background:**

Calmangafodipir (CaM, PledOx) demonstrated efficacy in preventing patient-reported chemotherapy-induced peripheral neuropathy (CIPN) in a randomized phase 2 study in patients with metastatic colorectal cancer. The Preventive Treatment of OxaLiplatin Induced peripherAl neuRopathy (POLAR) program aimed to assess efficacy and safety of CaM in the prevention of CIPN in patients treated with oxaliplatin in adjuvant (POLAR-A, ClinicalTrials.gov.NCT04034355) or metastatic (POLAR-M, ClinicalTrials.gov.NCT03654729) settings.

**Methods:**

Two randomized, placebo-controlled phase 3 trials investigated patient-reported, moderate-to-severe CIPN 9 months after beginning folinic acid, 5-fluorouracil, and oxaliplatin therapy with or without CaM. In POLAR-A, patients with stage III or high-risk stage II colorectal cancer were randomly assigned 1:1 to receive CaM 5 μmol/kg or placebo. In POLAR-M, patients with metastatic colorectal cancer were randomly assigned 1:1:1 to receive CaM 5 μmol/kg, CaM 2 μmol/kg, or placebo.

**Results:**

POLAR-A (n = 301) and POLAR-M (n = 291) were terminated early following unexpected hypersensitivity reactions in CaM-treated patients. In a combined analysis of month 9 CIPN (primary endpoint) data from both trials (CaM 5 μmol/kg, n = 175; placebo, n = 176), 54.3% of patients in the CaM group had moderate-to-severe CIPN compared with 40.3% in the placebo group. The estimated relative risk for moderate-to-severe CIPN at month 9 was 1.37 (95% confidence interval = 1.01 to 1.86; *P* = .045). A higher proportion of patients experienced serious hypersensitivity reactions across both trials with CaM treatment (3.6%) than with placebo (0.8%).

**Conclusion:**

The POLAR clinical studies failed to meet their primary endpoint. These results highlight the challenges of targeting oxidative stress for preventing CIPN in both the adjuvant and metastatic settings.

In colorectal cancer (CRC), oxaliplatin is used in combination with a fluoropyrimidine as adjuvant treatment to increase long-term survival and to improve efficacy in metastatic CRC (mCRC) (1-3). Oxaliplatin causes chronic chemotherapy-induced peripheral neuropathy (CIPN) in a cumulative dose-dependent manner (4,5). Chronic CIPN may be long-lasting and can severely impact quality of life (6,7). As there are no effective preventative or therapeutic treatments for oxaliplatin-induced CIPN, dose reduction or discontinuation remains the only strategy to prevent chronic CIPN (8,9).

Mitochondrial dysfunction and oxidative stress are key factors in the pathophysiology of CIPN (10). Clinical and preclinical data suggest that calmangafodipir (CaM; [Ca_0.8_, Mn_0.2_]Na_3_DPDP; PledOx) could be an efficacious inhibitor of CIPN and other conditions caused by cellular oxidative stress without interfering negatively with the antitumor activity of chemotherapy (11,12). CaM is a strong iron chelator and mimics the activity of manganese superoxide dismutase, inhibiting the formation of reactive oxygen species (12). A Double Blinded Randomised Three-Armed Phase II Trial of PledOx in Two Different Doses in Combination With FOLFOX6 Compared to Placebo + FOLFOX6 In PAtieNTs With Advanced Metastatic Colorectal (Stage IV) Cancer (PLIANT) was a randomized phase 2 study (n = 173, ClinicalTrials.gov.NCT01619423) (13) in which CaM reduced patient-reported symptoms of CIPN compared with placebo in patients with mCRC treated with modified folinic acid, 5-fluorouracil, and oxaliplatin (mFOLFOX6). Furthermore, CaM was well tolerated, with adverse events (AEs) of similar frequency and severity to those reported with placebo, with no detectable reduction in chemotherapy efficacy.

Based on the results of PLIANT, 2 large, independent phase 3 studies (Preventive Treatment of OxaLiplatin Induced peripherAl neuRopathy; POLAR) were conducted to assess efficacy and safety of CaM in preventing moderate-to-severe CIPN in the adjuvant (POLAR-A, ClinicalTrials.gov.NCT04034355) and metastatic (POLAR-M, ClinicalTrials.gov.NCT03654729) CRC settings.

## Methods

### Study Overview

The phase 3 POLAR program consisted of 2 randomized, multicenter, double-blind placebo-controlled studies. POLAR-A was conducted at 64 sites in 10 countries (Belgium, Czech Republic, France, Germany, Italy, Japan, South Korea, Spain, Taiwan, and the United Kingdom) and POLAR-M at 77 sites in 13 countries (same as POLAR-A as well as Hong Kong, Hungary, and the United States). Protocols and amendments were approved by an ethics committee or institutional review board for each site and the relevant national authorities. Studies were carried out in accordance with Good Clinical Practice guidelines and the Declaration of Helsinki and overseen by an independent data safety monitoring board (DSMB). All participants provided written informed consent.

On March 1, 2020, recruitment of patients in the POLAR program was put on hold, and no further study drug was administered. This decision followed recommendations by the French regulatory authority (L'Agence nationale de sécurité du médicament et des produits de santé) and the US Food and Drug Administration owing to 4 observed seizure events. The DSMB and an additional independent external evaluation judged that these events were not related to CaM. The occurrence of severe hypersensitivity reactions, primarily observed after repeated dosing, resulted in the termination of the program on April 6, 2020, following a recommendation from the DSMB. Though no further study drug was administered, all patients continued to have follow-up visits until 9-month data collection for the primary endpoint was completed. The data cutoff date for both studies was August 31, 2020.

### Participants

For POLAR-A, eligible patients were aged 18 years or older with pathologically confirmed CRC adenocarcinoma stage II or III, had undergone curative surgical resection within 12 weeks before random assignment, and were eligible for up to 6 months of oxaliplatin-based chemotherapy. For POLAR-M, participants were aged 18 years or older with nonresectable metastatic, pathologically confirmed adenocarcinoma of the colon or rectum. They had to be scheduled for at least 3 months of first-line oxaliplatin-based chemotherapy. In both studies, patients had an Eastern Cooperative Oncology Group performance status of 0 or 1 and no pathological findings from a neurological examination before oxaliplatin treatment. Further details on inclusion and exclusion criteria are presented in the Supplementary Methods (available online). Demographic information (see Table 1) was self-reported or investigator observed.

**Table 1.** POLAR: patient demographics and patient characteristics (combined mITT set)

Characteristic	CaM 5 μmol/kg (n = 216)	Placebo (n = 218)	Total (N = 434)
Age, y			
Median (range)	64.0 (25-87)	64.0 (31-84)	64.0 (25-87)
Sex, No. (%)^a^			
Female	101 (46.8)	89 (40.8)	190 (43.8)
Male	115 (53.2)	129 (59.2)	244 (56.2)
Race, No. (%)			
Asian	75 (34.7)	75 (34.4)	150 (34.6)
Black or African American	2 (0.9)	0 (0.0)	2 (0.5)
Native Hawaiian or other Pacific Islander	1 (0.5)	0 (0.0)	1 (0.2)
Other^b^	1 (0.5)	1 (0.5)	2 (0.5)
Unknown^c^	19 (8.8)	18 (8.3)	37 (8.5)
White	118 (54.6)	124 (56.9)	242 (55.8)
BMI, kg/m^2^			
Median (range)	24.2 (16.9-40.3)	24.0 (16.7-41.9)	24.2 (16.7-41.9)
ECOG performance status, No. (%)^d^			
0	118 (80.3)	122 (81.3)	240 (80.8)
1	29 (19.7)	28 (18.7)	57 (19.2)

a Reported by investigator. BMI = body mass index; CaM = calmangafodipir; ECOG = Eastern Cooperative Oncology Group; mITT = modified intention-to-treat; POLAR = Preventive Treatment of OxaLiplatin Induced peripherAl neuRopathy.

b “Other” included one mixed White and Asian and one Latino.

c Race is not allowed to be recorded in France.

d ECOG performance status: 0 = fully active, able to carry on all predisease performance without restriction; 1 = restricted in physically strenuous activity but ambulatory and able to carry out work of a light or sedentary nature (eg, light housework, office work).

### Study Design

**Figure 1.**
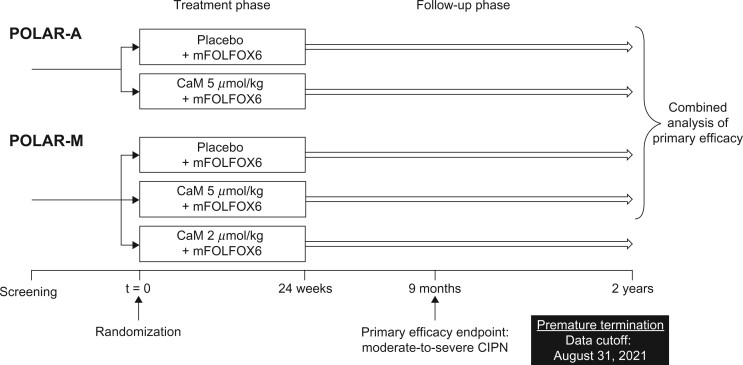
Study design. CaM = calmangafodipir; CIPN = chemotherapy-induced peripheral neuropathy; mFOLFOX6 = modified folinic acid, 5-fluorouracil, and oxaliplatin chemotherapy regimen; POLAR-A = Preventive Treatment of OxaLiplatin Induced peripherAl neuRopathy in adjuvant setting; POLAR-M = Preventive Treatment of OxaLiplatin Induced peripherAl neuRopathy in metastatic setting.

Both studies consisted of a screening period (up to 28 days), a treatment phase (24 weeks), and a follow-up phase (see Figure 1). The follow-up phase was planned to span 2 years from the first dose of study treatment for POLAR-A and 3 years for POLAR-M.

In POLAR-A, patients were randomly assigned 1:1 to receive CaM 5 μmol/kg or placebo (0.9% sodium chloride) as an intravenous infusion. Initially, the study treatment was given as a 5-minute infusion, administered 10 minutes before each mFOLFOX6 cycle, every 2 weeks for 12 cycles. Protocol amendments to both studies later changed the infusion duration to 10 minutes, administered 15 minutes before each cycle (Supplementary Table 1, available online). Patients in POLAR-A were assessed for efficacy and safety every 3 months up to month 12, then every 6 months to the end of follow-up.

In POLAR-M, patients were randomly assigned 1:1:1 to receive CaM 2 μmol/kg, CaM 5 μmol/kg, or placebo in addition to mFOLFOX6, following the protocol stated above. Patients in POLAR-M were assessed for efficacy and safety every 3 months from the first dose of study treatment.

After study drug dosing was suspended (January 23, 2020, in the United States and March 1, 2020, in the remaining 12 participating countries), patients who were still in the treatment phase had an end-of-treatment visit on day 14 of the last mFOLFOX6 cycle before August 31, 2020. Patients in the follow-up phase by August 31, 2020, had an end-of-study visit within 1 week of the end of August. No patients completed either study according to the planned protocol. Details of random assignment, blinding, study treatment, premedication, and chemotherapy administration can be found in the Supplementary Methods and Supplementary Tables 1 and 2 (available online).

### Outcomes

The primary efficacy endpoint was the proportion of patients with chronic CIPN at month 9, defined as a score of 3 or 4 in at least 1 of the first 4 items of the Functional Assessment of Cancer Therapy/Gynecologic Oncology Group-Neurotoxicity 13-item subscale (FACT/GOG-NTX-13) (14), targeting numbness, tingling, or discomfort in hands and/or feet. The FACT/GOG-NTX-13 was assessed within 72 hours before every mFOLFOX6 infusion during the treatment phase and at each assessment visit. Secondary efficacy endpoints evaluated at month 9 included cumulative dose of oxaliplatin and change from baseline in cold sensitivity, vibration sensitivity, functional impairment in the nondominant hand (measured using the grooved pegboard test), and pain in hands and feet. Full details can be found in the Supplementary Methods (available online).

Safety assessments included treatment-emergent AEs, laboratory analyses, Eastern Cooperative Oncology Group performance status, electrocardiograms, tumor and disease evaluations (disease-free survival [DFS] in POLAR-A and progression-free survival [PFS] and overall survival [OS] in POLAR-M), brain magnetic resonance imaging, and neurological examination. AE severity was graded using the Common Terminology Criteria for Adverse Events version 4.03, and relationship to study treatment was classified by the investigator.

### Statistical Analysis

With 112 patients per group, each POLAR study was estimated to have 91% power to detect a reduction vs placebo (improvement) from 40% to 20% (odds ratio [OR] = 0.375) in the primary endpoint using a 2-sided test controlled at the 0.05 type I error rate. A total of 140 patients per arm was planned to account for 20% dropout. Owing to the premature termination of the studies, POLAR-M was not fully recruited, and not all patients in either study had sufficient dosing of study treatment to assess primary efficacy. Therefore, a combined analysis of the primary endpoint was conducted across the 2 studies (see Figure 1) based on a modified intention-to-treat (mITT) analysis set. This set included patients who were eligible for at least 3 months of study treatment and who had at least 1 assessment for efficacy or a 3-month assessment visit prior to March 1, 2020, or received the sixth cycle of study drug after March 1, 2020. The primary endpoint was analyzed using the Cochran–Mantel–Haenszel estimate of the relative risk (RR) of moderate-to-severe CIPN. Treatment effect was assessed by estimating the ratio of the incidence of CIPN in patients receiving CaM 5 μmol/kg vs placebo, along with its 95% confidence interval (CI) and corresponding *P* value. The Cochran–Mantel–Haenszel estimate was adjusted for cumulative exposure to oxaliplatin and stratified by study and region.

Secondary efficacy endpoints were assessed in the mITT set by analysis of variance or covariance models using study, region (Asia or non-Asia), and treatment as factors. The safety analysis set comprised all randomly assigned patients who received at least 1 dose of study treatment. PFS, OS, and DFS data were analyzed by a Cox proportional hazards model, using region (Asia or non-Asia) and treatment as fixed factors, and depicted graphically as Kaplan–Meier plots. Schoenfeld global test was used to evaluate the proportional hazards assumption for the Cox regression.

## Results

### Patients

**Figure 2.**
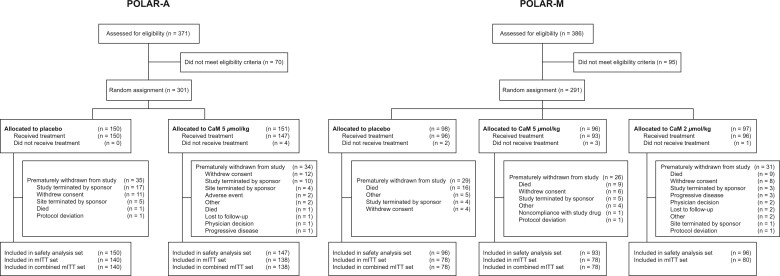
Patient flow. CaM = calmangafodipir; mITT = modified intention-to-treat; POLAR-A = Preventive Treatment of OxaLiplatin Induced peripherAl neuRopathy in adjuvant setting; POLAR-M = Preventive Treatment of OxaLiplatin Induced peripherAl neuRopathy in metastatic setting.

Between October 1, 2018, and March 1, 2020, 757 patients were screened for eligibility (Figure 2). In POLAR-A, 301 were randomly assigned, and 297 (98.7%) received at least 1 dose of study treatment. Of these patients, 34.0% in the CaM group and 38.0% in the placebo group received 12 cycles of study treatment. In POLAR-M, 291 patients were randomly assigned, and 285 (97.9%) received at least 1 dose of study treatment. Of these patients, 37.6% in the CaM 5 μmol/kg group, 36.5% in the CaM 2 μmol/kg group, and 29.2% in the placebo group received 12 cycles of study treatment. For the combined mITT set, 434 (87.7%) of 495 randomly assigned patients were included (216 in the CaM 5 μmol/kg group, 218 in the placebo group). Baseline characteristics were similar between treatment groups in each study and the combined mITT set (POLAR-A, Supplementary Table 3; POLAR-M, Supplementary Table 4, available online; combined set, Table 1).

### Efficacy

**Figure 3.**
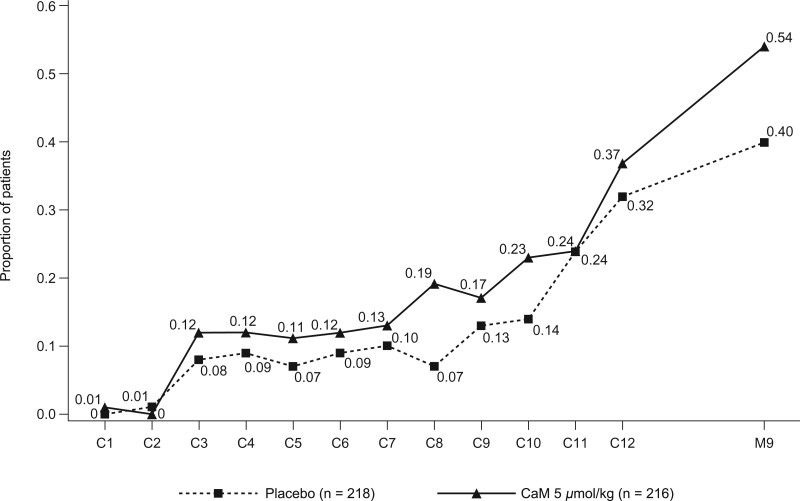
Proportion of patients in the POLAR studies with moderate-to-severe chemotherapy-induced peripheral neuropathy by treatment and visit (combined mITT set). These patients had a score of 3 or 4 on at least 1 of the first 4 items of the Functional Assessment of Cancer Therapy/Gynecologic Oncology Group-Neurotoxicity 13-item subscale at each treatment visit (cycle) and at 9 months (primary endpoint). C = cycle; CaM = calmangafodipir; mITT = modified intention-to-treat; POLAR = Preventive Treatment of OxaLiplatin Induced peripherAl neuRopathy.

The combined primary efficacy analysis included 351 patients for whom month 9 CIPN data were available (CaM 5 μmol/kg, n = 175; placebo, n = 176). An increased risk of patient-reported moderate-to-severe CIPN at month 9 was observed with CaM 5 μmol/kg compared with placebo (RR = 1.37, 95% CI = 1.01 to 1.86; *P* = .045; Table 2). The proportion of patients with moderate-to-severe CIPN generally increased over time and was higher in the CaM group than in the placebo group at most time points (Figure 3). This overall finding was driven by POLAR-A, in which the proportion of patients with CIPN at month 9 was statistically significantly higher in the CaM group than in the placebo group (RR = 1.52, 95% CI = 1.05 to 2.21; *P* = .03; Table 2). In POLAR-M, more patients in the 2 μmol/kg group than in the 5 μmol/kg or placebo groups reported moderate-to-severe CIPN at month 9 (Table 2). FACT–GOG-NTX-4 subscale results for each study are shown in Supplementary Figures 1 and 2 (available online).

**Table 2.** Analysis of moderate-to-severe CIPN at month 9 (mITT set)

Patient group	No.	Event rate^a,^^b^ (95% CI)	Relative risk vs placebo^c^ (95% CI)	*P*
Combined				
CaM 5 μmol/kg	175	0.548 (0.448 to 0.670)	1.37 (1.01 to 1.86)	.045
Placebo	176	0.400 (0.317 to 0.504)	—	—
POLAR-M				
CaM 5 μmol/kg	55	0.485 (0.332 to 0.707)	1.10 (0.64 to 1.89)	.74
Placebo	57	0.443 (0.299 to 0.655)	—	—
CaM 2 μmol/kg	54	0.590 (0.415 to 0.839)	1.38 (0.82 to 2.34)	.23
Placebo	57	0.426 (0.288 to 0.631)	—	—
POLAR-A				
CaM 5 μmol/kg	120	0.577 (0.455 to 0.732)	1.52 (1.05 to 2.21)	.03
Placebo	119	0.379 (0.284 to 0.507)	—	—

a Based on Cochran–Mantel–Haenszel analysis adjusted for region (Asia or non-Asia) and cumulative dose of oxaliplatin. CaM = calmangafodipir; CI = confidence interval; CIPN = chemotherapy-induced peripheral neuropathy; mITT = modified intention-to-treat; POLAR-A = Preventive Treatment of OxaLiplatin Induced peripherAl neuRopathy in adjuvant setting; POLAR-M = Preventive Treatment of OxaLiplatin Induced peripherAl neuRopathy in metastatic setting.

b Estimates of event rates per treatment arm of patients with moderate-to-severe CIPN according to the first 4 items of the Functional Assessment of Cancer Therapy/Gynecologic Oncology Group-Neurotoxicity 13-item subscale, targeting numbness, tingling, or discomfort in hands and/or feet 9 months after first dose of study treatment for the observed mean cumulative dose of oxaliplatin (mg/m^2^).

c Relative risk of the estimated event rate.

Among patients with mCRC, development of CIPN in the CaM group vs the placebo group was increased in Asian (RR = 1.30, 95% CI = 0.62 to 2.76; *P* = .49) but not in non-Asian (RR = 0.89, 95% CI = 0.40 to 2.00; *P* = .79) participants, though neither difference was statistically significant (Supplementary Table 5, available online). In a post hoc analysis of the Caucasian POLAR-M population (n = 47), the relative risk for the CaM group compared with the placebo group was 0.72 (95% CI = 0.30 to 1.73; *P* = .46).

In POLAR-A, cumulative oxaliplatin dose 9 months after the first dose of study drug was similar across treatment groups (Supplementary Table 6, available online). In POLAR-M, the least-squares mean cumulative oxaliplatin dose was numerically higher in the CaM 5 μmol/kg (804 mg/m^2^, 95% CI = 734 to 873) and 2 μmol/kg (781 mg/m^2^, 95% CI = 712 to 850) groups than in the placebo group (765 mg/m^2^, 95% CI = 695 to 834), but neither difference was statistically significant. Post hoc analysis showed that a statistically significantly higher proportion of patients in the CaM 5 μmol/kg group than in the placebo group in POLAR-M completed 12 cycles of both oxaliplatin and study treatment (24.4% vs 10.3%, *P* = .03; Supplementary Figure 3, available online).

Functional impairment in the nondominant hand, as assessed by time to complete the grooved pegboard test, increased statistically significantly more from baseline to month 9 with CaM 5 μmol/kg than with placebo (treatment difference vs placebo, 10.15 seconds, 95% CI = 0.20 to 20.10; *P* = .046; Supplementary Table 7, available online); however, the change from baseline in each treatment group was not considered clinically meaningful. There were no other statistically significant differences between CaM and placebo for any of the secondary efficacy endpoints, including cold sensitivity.

## Safety

### POLAR-A

In POLAR-A, 99.3% (n = 146) of patients in the CaM group and 97.3% (n = 146) in the placebo group experienced an AE (Table 3). Treatment-related AEs and treatment-related serious AEs (SAEs) were experienced by 24.5% (n = 36) and 5.4% (n = 8) of patients in the CaM group and 19.3% (n = 29) and 0% of patients in the placebo group, respectively (Tables 3 and 4). The most frequent treatment-related AEs were diarrhea, nausea, and fatigue. No difference in DFS was observed for CaM vs placebo (Supplementary Figure 4, available online).

**Table 3.** Summary of AEs in POLAR-A and POLAR-M (SAF)

AEs	POLAR-A		POLAR-M		
	CaM 5 μmol/kg (n = 147) No. (%)	Placebo (n = 150) No. (%)	CaM 5 μmol/kg (n = 93) No. (%)	CaM 2 μmol/kg (n = 96) No. (%)	Placebo (n = 96) No. (%)
Any AE	146 (99.3)	146 (97.3)	91 (97.8)	93 (96.9)	95 (99.0)
Any treatment-related AE^a^	36 (24.5)	29 (19.3)	31 (33.3)	18 (18.8)	17 (17.7)
Possibly related	26 (17.7)	17 (11.3)	15 (16.1)	11 (11.5)	8 (8.3)
Probably related	12 (8.2)	12 (8.0)	12 (12.9)	10 (10.4)	8 (8.3)
Definitely related	7 (4.8)	6 (4.0)	12 (12.9)	5 (5.2)	4 (4.2)
Any SAE	20 (13.6)	20 (13.3)	21 (22.6)	27 (28.1)	24 (25.0)
Any treatment-related SAE	8 (5.4)	0 (0.0)	2 (2.2)	2 (2.1)	2 (2.1)
SAEs leading to study treatment withdrawal	9 (6.1)	4 (2.7)	4 (4.3)	4 (4.2)	4 (4.2)
SAEs leading to death	1 (0.7)	0 (0.0)	3 (3.2)	2 (2.1)	3 (3.1)
AEs of special interest					
Convulsions	1 (0.7)	1 (0.7)	1 (1.1)	0 (0.0)	0 (0.0)
Neuropathy	109 (74.1)	105 (70.0)	67 (72.0)	72 (75.0)	68 (70.8)
Hypersensitivity^b^	39 (26.5)	39 (26.0)	29 (31.2)	27 (28.1)	30 (31.3)
Chemotherapy related	142 (96.6)	146 (97.3)	90 (96.8)	87 (90.6)	92 (95.8)

^a^ An AE or SAE was considered treatment related if it was registered by the investigator as being “possibly,” “probably,” or “definitely” related to the study treatment. AE = adverse event; CaM = calmangafodipir; POLAR-A = Preventive Treatment of OxaLiplatin Induced peripherAl neuRopathy in adjuvant setting; POLAR-M = Preventive Treatment of OxaLiplatin Induced peripherAl neuRopathy in metastatic setting; SAE = serious adverse event; SAF = safety analysis set.

b The most common hypersensitivity AEs were rash, infusion-related reaction, and drug hypersensitivity.

**Table 4.** SAEs in POLAR-A (SAF)

SAEs	CaM 5 μmol/kg (n = 147) No. (%)	Placebo (n = 150) No. (%)
Any SAE	20 (13.6)	20 (13.3)
Treatment-related SAEs^a^	8 (5.4)	0 (0.0)
Infusion-related reaction	3 (2.0)	0 (0.0)
Anaphylactic reaction	1 (0.7)	0 (0.0)
Drug hypersensitivity	1 (0.7)	0 (0.0)
Cerebral infarction	1 (0.7)	0 (0.0)
Generalized tonic-clonic seizure	1 (0.7)	0 (0.0)
Vena cava thrombosis	1 (0.7)	0 (0.0)
SAEs leading to study treatment withdrawal	9 (6.1)	4 (2.7)
Infusion-related reactions	3 (2.0)	0 (0.0)
Anaphylactic reaction	1 (0.7)	0 (0.0)
Drug hypersensitivity	1 (0.7)	0 (0.0)
Cerebral infarction	1 (0.7)	0 (0.0)
Tonic-clonic seizure	1 (0.7)	0 (0.0)
Pulmonary embolism	1 (0.7)	1 (0.7)
Acute kidney injury	1 (0.7)	0 (0.0)
Myocardial infarction	0 (0.0)	1 (0.7)
Administration site cellulitis	0 (0.0)	1 (0.7)
Device-related infection	0 (0.0)	1 (0.7)
SAEs leading to death^b^	1 (0.7)	0 (0.0)
Tonic-clonic seizure	1 (0.7)	0 (0.0)

a An SAE was considered treatment related if it was registered by the investigator as being “possibly,” “probably,” or “definitely” related to the study treatment. CaM = calmangafodipir; POLAR-A = Preventive Treatment of OxaLiplatin Induced peripherAl neuRopathy in adjuvant setting; SAE = serious adverse event; SAF = safety analysis set.

b The SAE leading to death was considered possibly related to the study treatment because of a temporal association. The patient had received 8 previous infusions without any major findings or concerns.

Hypersensitivity SAEs were experienced by 6 patients in the CaM 5 μmol/kg group (Supplementary Table 8, available online). Three patients experienced infusion-related reactions, 2 had anaphylactic reactions (1 unlikely to be related to treatment), and 1 had drug hypersensitivity. One patient in the placebo group experienced a hypersensitivity SAE of moderate pneumonitis that was possibly related to treatment. All patients recovered from the hypersensitivity SAE except for the individual receiving placebo (pneumonitis SAE).

Blood manganese values increased in the CaM group relative to the placebo group. Increases were reported as AEs for only 1 patient in each treatment group, and the events did not lead to study treatment withdrawal. No further safety concerns were raised from reviewing hematology and biochemistry test values. There were no clinically meaningful findings in vital signs, weight and body mass index, physical examination assessments, or electrocardiograms.

### POLAR-M

In POLAR-M, 97.8% (n = 91) of patients in the CaM 5 μmol/kg group, 96.9% (n = 93) in the CaM 2 μmol/kg group, and 99.0% (n = 95) in the placebo group experienced an AE (Table 3). The proportion of patients experiencing a treatment-related AE was 33.3% (n = 31) in the CaM 5 μmol/kg group, 18.8% (n = 18) in the CaM 2 μmol/kg group, and 17.7% (n = 17) in the placebo group. The most frequent treatment-related AEs were neutropenia, diarrhea, nausea, fatigue, peripheral neuropathy, and rash. Numbers of treatment-related SAEs were low and were comparable across treatment groups (Table 5). No differences in PFS or OS were observed for either CaM group vs placebo (Supplementary Figures 5 and 6, available online).

**Table 5.** SAEs in POLAR-M (SAF)

SAEs	CaM 5 μmol/kg (n = 93) No. (%)	CaM 2 μmol/kg (n = 96) No. (%)	Placebo (n = 96) No. (%)
Any SAE	21 (22.6)	27 (28.1)	24 (25.0)
Treatment-related SAEs^a^	2 (2.2)	2 (2.1)	2 (2.1)
Infusion-related reaction	0 (0.0)	1 (1.0)	0 (0.0)
Hyperkalemia	0 (0.0)	0 (0.0)	1 (1.0)
Hypokalemia	1 (1.1)	0 (0.0)	0 (0.0)
Altered state of consciousness	0 (0.0)	1 (1.0)	0 (0.0)
Presyncope	0 (0.0)	1 (1.0)	0 (0.0)
Nausea	0 (0.0)	1 (1.0)	0 (0.0)
Vomiting	0 (0.0)	1 (1.0)	0 (0.0)
Renal failure	0 (0.0)	0 (0.0)	1 (1.0)
Febrile neutropenia	1 (1.1)	0 (0.0)	0 (0.0)
Ovarian vein thrombosis	1 (1.1)	0 (0.0)	0 (0.0)
SAEs leading to study treatment withdrawal	4 (4.3)	4 (4.2)	4 (4.2)
Drug hypersensitivity	1 (1.1)	0 (0.0)	0 (0.0)
Cardiogenic shock	1 (1.1)	0 (0.0)	0 (0.0)
Peritonitis	0 (0.0)	0 (0.0)	2 (2.1)
Pneumonia	0 (0.0)	1 (1.0)	0 (0.0)
Toxicity to various agents	0 (0.0)	0 (0.0)	1 (1.0)
Completed suicide	0 (0.0)	0 (0.0)	1 (1.0)
Death	0 (0.0)	1 (1.0)	0 (0.0)
Euthanasia	0 (0.0)	1 (1.0)	0 (0.0)
Altered state of consciousness	0 (0.0)	1 (1.0)	0 (0.0)
Respiratory distress	0 (0.0)	1 (1.0)	0 (0.0)
Intestinal occlusion	1 (1.1)	0 (0.0)	0 (0.0)
Neutropenic colitis	1 (1.1)	0 (0.0)	0 (0.0)
SAEs leading to death^b^	3 (3.2)	2 (2.1)	3 (3.1)
Coronavirus infection	1 (1.1)	0 (0.0)	0 (0.0)
Intestinal obstruction	1 (1.1)	0 (0.0)	0 (0.0)
Cardiogenic shock	1 (1.1)	0 (0.0)	0 (0.0)
Euthanasia	0 (0.0)	1 (1.0)	0 (0.0)
Death, unknown	0 (0.0)	1 (1.0)	0 (0.0)
Completed suicide	0 (0.0)	0 (0.0)	1 (1.0)
Peritonitis	0 (0.0)	0 (0.0)	1 (1.0)
Toxicity to various agents	0 (0.0)	0 (0.0)	1 (1.0)

a An SAE was considered treatment related if it was registered by the investigator as being “possibly,” “probably,” or “definitely” related to the study treatment. CaM = calmangafodipir; POLAR-M = Preventive Treatment of OxaLiplatin Induced peripherAl neuRopathy in metastatic setting; SAE = serious adverse event; SAF = safety analysis set.

b In all cases, the SAE leading to death was considered not related or unlikely related to the study treatment.

Hypersensitivity SAEs were experienced by 3 patients in the CaM 5 μmol/kg group (hypersensitivity, drug hypersensitivity, anaphylactic shock), 3 patients in the CaM 2 μmol/kg group (pneumonitis, infusion-related reaction, urticaria), and 1 patient in the placebo group (urticaria) (Supplementary Table 9, available online). Except for the infusion-related reaction in the 2 μmol/kg group, all other hypersensitivity SAEs were deemed either not related or unlikely related to treatment. All patients recovered from the hypersensitivity SAE.

Increased blood manganese levels were found in the CaM groups compared with the placebo group. These were reported as AEs for a small number (CaM 5 μmol/kg, n = 3; CaM 2 μmol/kg, n = 1) of patients; in 1 patient, the AE led to interruption of study treatment dosing. No other clinically meaningful findings in safety parameters were identified.

## Discussion

The POLAR clinical program assessed the efficacy and safety of CaM in preventing oxaliplatin-associated CIPN in patients with CRC in the adjuvant and metastatic settings. Owing to early termination of the studies, the primary efficacy analysis of moderate-to-severe CIPN at month 9 was performed using combined data from POLAR-A and POLAR-M. The POLAR program did not meet its primary endpoint, with CaM treatment increasing the risk of CIPN vs placebo at month 9. This is yet another in a long list of studies that has failed to demonstrate meaningful prevention of oxaliplatin-induced CIPN. The first American Society of Clinical Oncology guideline from 2014 could not recommend any agent for prevention of CIPN, despite reviewing 48 randomized controlled trials covering a range of mechanisms (15). In the updated guideline from 2020, review of another 28 randomized controlled trials reconfirmed the conclusion that no agent could be recommended (9). A recent study of riluzole also failed to demonstrate prevention (16). Interestingly, that study, similar to the current study, found that patient-reported FACT/GOG-NTX scores were statistically significantly worse in the active arm.

CaM previously reduced patient-reported, but not physician-reported, symptoms of CIPN vs placebo in patients with mCRC in the phase 2 PLIANT study, which did not meet its primary endpoint (13). The addition of patients undergoing adjuvant treatment in the POLAR program may have impacted the observed results. CaM produces a bell-shaped dose-response curve on relevant CIPN parameters in preclinical models (17), similar to endogenous superoxide dismutase (18). Tumorigenesis is associated with increasing levels of oxidative stress in the tumor (19); potentially, this is also the case in peripheral nerves. Thus, an increased tumor burden in patients with mCRC, driving higher levels of oxidative stress, could result in these individuals responding differently to a given dose of CaM to patients in the adjuvant setting. The POLAR program also included Asian and non-Asian patients, whereas the PLIANT study was conducted in a predominantly Caucasian population. Analyses of the Asian and non-Asian patients in POLAR-M indicate potential differences in response to CaM treatment.

The timing of CaM administration relative to oxaliplatin may result in unfavorable metal-based redox interactions driving further cellular oxidative and nitrosative stress (20). It is important to note that in the POLAR program, as in the PLIANT study and preclinical studies (17), CaM was initially administered as a 5-minute infusion delivered 10 minutes before oxaliplatin treatment. In January 2020, the CaM administration schedule was amended to specify a 10-minute infusion delivered 15 minutes before oxaliplatin, with the aim of further minimizing hypersensitivity reactions. Therefore, the timing of CaM administration may not satisfactorily explain the divergent primary efficacy results between the PLIANT and POLAR studies.

The serious hypersensitivity reactions observed, particularly after repeated CaM dosing, were unexpected and deemed significant enough for the DSMB to recommend terminating both studies after unblinding. Hypersensitivity SAEs were more frequent in patients receiving CaM than in those receiving placebo (12 patients in the CaM groups combined [total n = 336] vs 2 in the placebo groups [total n = 246]). These SAEs were typically seen at CaM and mFOLFOX6 cycle 6 or later, apart from 1 reaction that occurred after the first dose of CaM. Although the origin of these reactions remains unclear, they share features with hypersensitivity to platinum drugs (21). Postponing CaM infusion until 30 minutes after oxaliplatin administration, as in a previous study of mangafodipir (22), may potentially reduce hypersensitivity.

A limitation of the POLAR program is the early termination, which resulted in insufficient data for the planned analyses, and data from the 2 studies were combined for assessment of the primary endpoint. Differences in racial demographics across studies may also have confounded study results. Premature termination also prevented any longer-term assessment of safety or efficacy.

In conclusion, the POLAR clinical studies failed to meet their primary endpoint, with CaM showing no benefit in preventing moderate-to-severe CIPN at month 9. In fact, CaM-treated patients showed statistically significantly increased risk of CIPN vs placebo. Furthermore, a higher number of patients experienced hypersensitivity SAEs with CaM than with placebo. These results once again highlight the challenges of preventing CIPN.

## Funding

The POLAR-A and POLAR-M trials were funded by Egetis Therapeutics AB (Stockholm, Sweden) and co-sponsored by Solasia Pharma K.K. (Tokyo, Japan) in Asia.

## Notes

**Role of the funder:** Egetis Therapeutics was involved in the study design and in the collection, analysis, interpretation, and reporting of the data. The study funder provided funding for medical writing assistance with manuscript preparation.

**Disclosures:** P Pfeiffer has received research funding, consulting fees, or honoraria from Taiho, Servier, Nordic Drugs, Shire, Merck, PledPharma, Isofol, Lilly, Roche, Merck-Serono, Amgen, and Celgene. M Lustberg has received consulting fees from AstraZeneca, Biotheranostics, Novartis, Pfizer, and PledPharma. J Näsström is an employee and shareholder of Egetis Therapeutics AB and is an inventor/co-inventor of Egetis patent applications/patents. S Carlsson was an employee of Egetis Therapeutics AB at the time of the POLAR clinical studies and is currently an employee of Calliditas Therapeutics. A Persson has received consulting fees from Egetis Therapeutics AB. F Nagahama is an employee of Solasia Pharma K.K. G Cavaletti has received consulting fees from Algo Therapeutics, Disarm Therapeutics, Egetis Therapeutics AB, Kedrion, Novartis, Seattle Genetics, and Toray. B Glimelius has received research funding from Amgen. K Muro has received research funding, consulting fees, or honoraria from Amgen, Astellas, AstraZeneca, Bayer, Bristol-Myers Squibb, Chugai, Daiichi Sankyo, Eisai, Eli Lilly, Merck Serono, Merck Sharp & Dohme, ONO Pharmaceutical, Parexel International, Pfizer, Sanofi, Solasia Pharma, Taiho, and Takeda.

**Author contributions:** Conceptualization and methodology: J Näsström, S Carlsson, A Persson, F Nagahama, G Cavaletti, B Glimelius, P Pfeiffer. Investigation: P Pfeiffer, M Lustberg, K Muro. Data curation: All authors. Formal analysis: A Persson. Writing–original draft: all authors. Writing–review and editing: all authors.

**Acknowledgements:** Medical writing support for the preparation of this manuscript was provided by Freyja McClenahan, PhD, and Sarah Graham, PhD, of PharmaGenesis London, London, UK, and was funded by Egetis Therapeutics.

**Prior presentations:** Muro K, Yamazaki K, Lee K-W, et al. Asian subgroup analysis in global POLAR program of calmangafodipir on top of mFOLFOX6 for the prevention of CIPN. Presented at the Japanese Society of Medical Oncology Annual Meeting, Kyoto, Japan, February 2022; Mini-Oral Session 36-4.

Qvortrup C, Muro K, Lustberg M, et al. The global POLAR program: top-line results of placebo-controlled studies of calmangafodipir on top of modified FOLFOX6 to prevent chemotherapy-induced peripheral neuropathy. *Ann Oncol.* 2021;32(suppl 3):S209-S210.

## Supplementary Material

pkac075_Supplementary_Data

## Data Availability

Data will be shared upon reasonable request to the corresponding author for research purposes, dependent upon the nature of the request, the merit of the proposed research, the availability of the data, and its intended use. Individual participant data from the clinical studies will be anonymized.
