# Supplementary material for: Calmangafodipir for Prevention of Oxaliplatin-Induced Peripheral Neuropathy: Two Placebo-Controlled, Randomized Phase 3 Studies (POLAR-A/POLAR-M)
Source: JNCI Cancer Spectr. 2022 Oct 29;6(6):pkac075. doi: 10.1093/jncics/pkac075 (PMC9678401; doi:10.1093/jncics/pkac075)
Supplement: pkac075_Supplementary_Data [file pkac075_supplementary_data.pdf]

Supplementary Materials

<u>Contents</u>

Supplementary Methods

Randomization and Masking

Inclusion and exclusion criteria

Efficacy endpoints

Supplementary References

Supplementary Table 1. Study treatment and premedications administration schedule

Supplementary Table 2. Chemotherapy administration schedule

Supplementary Table 3. POLAR-A: patient demographics and characteristics at baseline

Supplementary Table 4. POLAR-M: patient demographics and characteristics at baseline

Supplementary Table 5. POLAR: analysis of moderate-to-severe CIPN at month 9 in Asian/ non-Asian subgroups (mITT)

Supplementary Table 6. Study treatment and oxaliplatin exposure (mITT) **Supplementary Table 7.** POLAR: change from baseline in time to complete the grooved pegboard test with the nondominant hand at 9 months (combined mITT)

Supplementary Table 8. POLAR-A: hypersensitivity SAEs

Supplementary Table 9. POLAR-M: hypersensitivity SAEs

Supplementary Figure 1. POLAR-A: FACT/GOG-NTX-4 subscale items (mITT)

Supplementary Figure 2. POLAR-M: FACT/GOG-NTX-4 subscale items (mITT)

Supplementary Figure 3. Proportion of patients completing 12 cycles of both oxaliplatin and study treatment (mITT)

Supplementary Figure 4. POLAR-A: Kaplan–Meier plot of disease-free survival (SAF)

Supplementary Figure 5. POLAR-M: Kaplan–Meier plot of progression-free survival (SAF)

Supplementary Figure 6. POLAR-M: Kaplan–Meier plot of overall survival (SAF)

Supplementary Methods

Randomization and Masking

Randomization was performed by blinded site staff using an interactive web response system (IWRS) within 3 days before cycle 1, day 1 of modified folinic acid/5-fluorouracil/oxaliplatin (mFOLFOX6) chemotherapy. Study treatment was prepared in an amber- colored syringe and administered using amber-colored tubing (or another blinding system) by study personnel who were either blinded or not involved in the study. Unblinding could be performed via the IWRS if it was deemed medically necessary by the investigator. On March 6, 2020, unblinding of Egetis Therapeutics' core study team was approved for 10 patients with hypersensitivity reactions or seizures. On June 10, 2020, unblinding was approved for 6 additional patients with allergic infusion reactions or seizures. Otherwise, blinding was maintained until database lock. Patients whose treatment was unblinded continued to be monitored in the studies.

Inclusion and exclusion criteria

POLAR-M

Inclusion Criteria

Patients were eligible for inclusion in the POLAR-M study if all the following inclusion criteria were met.

1. Signed informed consent form received before any study-related assessments began and patient willing to follow all study procedures.
2. Male or female patients aged ≥18 years.
3. Nonresectable metastatic (stage IV) colorectal cancer (CRC), pathologically confirmed adenocarcinoma of the colon or rectum.
4. No prior systemic chemotherapy and/or biological/targeted therapy for metastatic CRC.
5. Measurable disease according to Response Evaluation Criteria in Solid Tumours (RECIST) 1.1 (1).
6. Patient with CRC indicated for at least 3 months of oxaliplatin-based chemotherapy (without any preplanned treatment breaks) and without pathological findings of a neurological examination performed before oxaliplatin treatment according to local practice.
7. Eastern Cooperative Oncology Group (ECOG) performance status of 0 or 1.
8. Adequate hematological parameters: hemoglobin concentration ≥100 g/L, absolute neutrophil count $\geq 1.5 \times 10^9$/L, platelet count $\geq 100 \times 10^9$/L.
9. Adequate renal function: creatinine clearance >50 mL/minute using the Cockcroft and Gault formula or measured.
10. Adequate hepatic function: total bilirubin level ≤1.5 × the upper limit of normal (ULN) (except in the case of known Gilbert's syndrome); aspartate aminotransferase (AST)

and alanine aminotransferase (ALT) ≤3 × ULN (AST and ALT ≤5 × ULN in the case of liver metastases).

11. Baseline blood manganese level <2.0 × ULN.
12. For patients with a history of diabetes mellitus, glycated hemoglobin ≤7%.
13. Negative pregnancy test for women of childbearing potential (WOCBP).
14. For men and WOCBP, use of adequate contraception (oral contraceptives, intrauterine device, or surgically sterile) while receiving study treatment and for at least 6 months after completion of study therapy.

Exclusion Criteria

Patients who met any of the following exclusion criteria were not eligible to participate in the POLAR-M study.

1. Any unresolved toxicity by National Cancer Institute-Common Terminology Criteria for Adverse Events (NCI-CTCAE) version 4.03 above grade 1 from previous anticancer therapy (including radiotherapy), except alopecia.
2. Any grade of neuropathy with any cause.
3. Any evidence of severe or uncontrolled systemic diseases (eg, unstable or uncompensated respiratory, cardiac, unresolved bowel obstruction, hepatic, or renal disease).
4. Chronic infection or uncontrolled serious illness causing immunodeficiency. Patients with a known history of chronic hepatitis B could be enrolled if they were asymptomatic and an acute and active hepatitis B virus infection could be excluded.
5. Any history of seizures.
6. A surgical incision that was not healed.
7. Significant hemorrhage (>30 mL/bleeding episode in the previous 3 months), hemoptysis (>5 mL of fresh blood in the previous 4 weeks), or thrombotic event (including transient ischemic attack) in the previous 12 months if the patient was

expected to receive antivascular endothelial growth factor/vascular endothelial growth factor receptor therapy.

8. Known hypersensitivity to any of the components of mFOLFOX6 and, if applicable, biological therapies that were to be used in conjunction with the chemotherapy regimen or any of the excipients of these products.
9. A history of other malignancies (except for adequately treated basal or squamous cell carcinoma or carcinoma in situ) within 5 years, unless the patient had been disease free for that other malignancy for at least 2 years.
10. Known dihydropyrimidine dehydrogenase deficiency.
11. Pre-existing neurodegenerative disease (eg, Parkinson's, Alzheimer's, Huntington's) or neuromuscular disorder (eg, multiple sclerosis, amyotrophic lateral sclerosis, polio, hereditary neuromuscular disease).
12. Major psychiatric disorder (major depression, psychosis), alcohol and/or drug abuse.
13. Patients with a history of, or a family history of, second- or third-degree atrioventricular block.
14. A history of a genetic or familial neuropathy.
15. Treatment with any investigational drug within 30 days before randomization.
16. Pregnancy, lactation, or reluctance to use contraception.
17. Any other condition that, in the opinion of the investigator, placed the patient at undue risk.
18. Previous exposure to mangafodipir or calmangafodipir.
19. Welders, mine workers, or other workers in occupations (current or past) where high manganese exposure was likely.

POLAR-A

Inclusion Criteria

Patients were eligible for inclusion in the POLAR-A study if all the following inclusion criteria were met.

1. Signed informed consent form received before any study-related assessments began and patient willing to follow all study procedures.
2. Male or female patients aged ≥18 years.
3. Pathologically confirmed adenocarcinoma of the colon or rectum including: stage III carcinoma (any T N1,2 M0) or stage II carcinoma (T3,4 N0 M0) (2).
4. The patient had undergone curative (R0) surgical resection within 12 weeks before randomization.
5. The patient had a postsurgical carcinoembryonic antigen level ≤1.5 × ULN (in current smokers, carcinoembryonic antigen level ≤2.0 × ULN was allowed).
6. No prior anticancer therapy for CRC except radiotherapy or concomitant chemoradiotherapy using a fluoropyrimidine alone for locoregional rectal cancer.
7. Patient with CRC indicated for up to 6 months of oxaliplatin-based chemotherapy and without pathological findings of a neurological examination performed before oxaliplatin treatment according to local practice.
8. ECOG performance status of 0 or 1.
9. Adequate hematological parameters: hemoglobin concentration ≥100 g/L, absolute neutrophil count ≥1.5 × 10^9/L, platelet count ≥100 × 10^9/L.
10. Adequate renal function: creatinine clearance >50 mL/minute using the Cockcroft and Gault formula or measured.
11. Adequate hepatic function: total bilirubin level ≤1.5 × ULN (except in the case of known Gilbert's syndrome); AST and ALT ≤3 × ULN.
12. Baseline blood manganese level <2.0 × ULN.
13. For patients with a history of diabetes mellitus, glycated hemoglobin ≤7%.
14. Negative pregnancy test for WOCBP.
15. For men and WOCBP, use of adequate contraception (oral contraceptives, intrauterine device, or surgically sterile) while receiving study treatment and for at least 6 months after completion of study therapy.

Exclusion Criteria

Patients who met any of the following exclusion criteria were not eligible to participate in the POLAR-A study.

1. Any evidence of metastatic disease.
2. Any unresolved toxicity by NCI-CTCAE version 4.03 above grade 1 from previous anticancer therapy (including radiotherapy), except alopecia.
3. Any grade of neuropathy with any cause.
4. Any evidence of severe or uncontrolled systemic diseases (eg, unstable or uncompensated respiratory, cardiac, unresolved bowel obstruction, hepatic, or renal disease).
5. Chronic infection or uncontrolled serious illness causing immunodeficiency. Patients with a known history of chronic hepatitis B could be enrolled if they were asymptomatic and an acute and active hepatitis B virus infection could be excluded.
6. Any history of seizures.
7. A surgical incision that was not healed.
8. Known hypersensitivity to any of the components of mFOLFOX6 and, if applicable, therapies that were to be used in conjunction with the chemotherapy regimen or any of the excipients of these products.
9. A history of other malignancies (except for adequately treated basal or squamous cell carcinoma or carcinoma in situ) within 5 years, unless the patient had been disease free for that other malignancy for at least 2 years.
10. Known dihydropyrimidine dehydrogenase deficiency.
11. Pre-existing neurodegenerative disease (eg, Parkinson’s, Alzheimer’s, Huntington’s) or neuromuscular disorder (eg, multiple sclerosis, amyotrophic lateral sclerosis, polio, hereditary neuromuscular disease).
12. Major psychiatric disorder (major depression, psychosis), alcohol and/or drug abuse.

13. Patients with a history of, or a family history of, second- or third-degree atrioventricular block.
14. A history of a genetic or familial neuropathy.
15. Treatment with any investigational drug within 30 days before randomization.
16. Pregnancy, lactation, or reluctance to use contraception.
17. Any other condition that, in the opinion of the investigator, placed the patient at undue risk.
18. Previous exposure to mangafodipir or calmangafodipir.
19. Welders, mine workers, or other workers in occupations (current or past) where high manganese exposure was likely.

Efficacy endpoints

- The primary efficacy endpoint was the proportion of patients with moderate-to-severe chemotherapy-induced peripheral neuropathy (CIPN) 9 months after the first dose of study treatment.
- Secondary efficacy endpoints were as follows:
  - the proportion of patients with mild-to-severe CIPN 9 months after the first dose of study treatment
  - the mean change from baseline in sensitivity to touching cold items on day 2, cycle 4 of mFOLFOX6 chemotherapy, as assessed by the Cold Sensitivity questionnaire (ordinal scale 0–10)
  - the mean cumulative dose of oxaliplatin administered per patient during mFOLFOX6 chemotherapy 9 months after the first dose of study treatment
  - the mean change from baseline in vibration sense (0–8 ordinal scale), on the lateral malleolus (left and right), using a graduated tuning fork, 9 months after the first dose of study treatment
  - the mean change from baseline in worst pain in hands or feet in the past week, using a numerical rating scale, 9 months after the first dose of study treatment
  - the mean change from baseline in the time (in seconds) to complete the grooved pegboard test with the nondominant hand 9 months after the first dose of study treatment
  - the proportion of patients with long-term moderate-to-severe CIPN 12 months after the first dose of study treatment.
- Exploratory effiacy endpoints were as follows:
  - 5-dimension 5 level EuroQol questionnaire score
  - health economic impact.

Supplementary References

1. Eisenhauer EA, Therasse P, Bogaerts J, et al. New response evaluation criteria in solid tumours: revised RECIST guideline (version 1.1). *Eur J Cancer.* 2009;45(2):228-47.
2. Brierley JD, Gospodarowicz MK, Wittekind C. *TNM classification of malignant tumours*: John Wiley & Sons; 2017.

Supplementary Table 1. Study treatment and premedications administration schedules

Arm	Dose[a]	Infusate/ preparation	Administration	Duration	Time point
A+B+C[a]	Diphenhydramine (1–2 mg/kg or 25–50 mg), ranitidine (50 mg)	Ranitidine diluted in 5% dextrose to a total volume of 20 mL	Slowly via i.v. in combination	Approx. 5 minutes	Approximately 5 minutes before study treatment
Before January 9, 2020					
A[b,c]	CaM 2 μmol/kg	Ready-to-use formulation. CaM was diluted in 0.9% NaCl to achieve the correct volume (dose) per body weight	i.v. infusion	Approx. 5 minutes	Approximately 10 minutes before the start of chemotherapy (day 1 of each cycle)
B[b]	CaM 5 μmol/kg				
C[b]	Placebo				
After January 9, 2020					
A[b,c]	CaM 2 μmol/kg	Ready-to-use formulation. CaM was diluted in 0.9% NaCl to achieve the correct volume (dose) per body weight	i.v. infusion	Approximately 10 minutes	Approximately 15 minutes before the start of chemotherapy (day 1 of each cycle)
B[b]	CaM 5 μmol/kg				
C[b]	Placebo				

Protocol amendments to both studies on January 9, 2020 changed the duration of study treatment infusion.
[a]Alternative products, doses, or ways of administration of antihistamines could be used, according to local clinical practice.
[b]A specific template for dose calculation was provided. All patients received 20 mL (ie, the volume of CaM/placebo was based on body weight).
[c]POLAR-M only.
CaM = calmangafodipir; i.v. = intravenous.

Supplementary Table 2. Chemotherapy administration schedules

Drug	Dose	Administration	Duration	Infusate/ preparation	Time point
Ondansetron[a]	8 mg[a]	i.v. injection	–	–	At least 30 minutes before the start of chemotherapy
Beta-methasone[a]	8 mg[a]	i.v. injection	–	–	At least 30 minutes before the start of chemotherapy
Oxaliplatin	85 mg/m^2	i.v. infusion	0–2 hours	0.5 L glucose 5%	Start of chemotherapy (regarded as time point 0)
Ca-levofolinate or	100 or 200 mg/m^2	i.v. infusion	0–2 hours	0.5 L glucose 5%	Start of chemotherapy (regarded as time point 0)
Ca-folinate	200 or 400 mg/m^2	i.v. infusion	0–2 hours	0.5 L glucose 5%	
5-FU	400 mg/m^2	i.v. bolus	–	Recommendation 50–100 mL NaCl 0.9%	Approximately 2 hours after time point 0
5-FU	2400 mg/m^2	i.v. continued infusion	2–28 hours	–	Approximately 2 hours after time point 0

In POLAR-M, the addition of an appropriate biological therapy (bevacizumab, panitumumab, cetuximab) was left to the discretion of the investigator.

[a]For antiemetics and corticosteroids, alternative doses or alternative products to those given above could be used, according to local clinical practice.

FU = fluorouracil; i.v. = intravenous.

Supplementary Table 3. POLAR-A: patient demographics and characteristics at baseline (SAF)

Characteristic	CaM 5 µmol/kg (n = 147)	Placebo (n = 150)	Total (n = 297)
Age, years			
Median (range)	64.0 (25–87)	64.5 (31–81)	64.0 (25–87)
Sex,[a] n (%)			
Female	69 (46.9)	61 (40.7)	130 (43.8)
Male	78 (53.1)	89 (59.3)	167 (56.2)
Race, n (%)			
Asian	43 (29.3)	43 (28.7)	86 (29.0)
Black or African American	1 (0.7)	0 (0.0)	1 (0.3)
Native Hawaiian or other Pacific Islander	1 (0.7)	0 (0.0)	1 (0.3)
Other[b]	1 (0.7)	1 (0.7)	2 (0.7)
Unknown[c]	14 (9.5)	13 (8.7)	27 (9.1)
White	87 (59.2)	93 (62.0)	180 (60.6)
BMI, kg/m^2			
Median (range)	24.2 (16.9–38.9)	23.6 (16.8–49.7)	24.2 (16.8–49.7)
ECOG performance status, n (%)			
0	118 (80.3)	122 (81.3)	240 (80.8)
1	29 (19.7)	28 (18.7)	57 (19.2)

[a]Reported by investigator.
[b]'Other' included White/Asian and Latino.
[c]Race is not allowed to be recorded in France.
ECOG performance status: 0 = fully active, able to carry on all predisease performance without restriction; 1 = restricted in physically strenuous activity but ambulatory and able to carry out work of a light or sedentary nature (eg, light housework, office work).
BMI = body mass index; CaM = calmangafodipir; ECOG = Eastern Cooperative Oncology Group; SAF = safety analysis set.

Supplementary Table 4. POLAR-M: patient demographics and characteristics at baseline (SAF)

Characteristic	CaM 5 µmol/kg (n = 93)	CaM 2 µmol/kg (n = 96)	Placebo (n = 96)	Total (n = 285)
Age, years				
Median (range)	63.0 (41–82)	64.5 (35–85)	64.0 (25–84)	64.0 (25–85)
Sex,[a] n (%)				
Female	43 (46.2)	36 (37.5)	40 (41.7)	119 (41.8)
Male	50 (53.8)	60 (62.5)	56 (58.3)	166 (58.2)
Race, n (%)				
Asian	40 (43.0)	41 (42.7)	39 (40.6)	120 (42.1)
Black or African American	1 (1.1)	0 (0.0)	1 (1.0)	2 (0.7)
Unknown[b]	7 (7.5)	6 (6.3)	5 (5.2)	18 (6.3)
White	45 (48.4)	49 (51.0)	51 (53.1)	145 (50.9)
BMI, kg/m^2				
Median (range)	24.2 (17.4–40.3)	23.6 (16.5–35.3)	24.0 (16.5–32.8)	24.0 (16.5–40.3)
ECOG performance status,[c] n (%)				
0	62 (66.7)	65 (67.7)	53 (56.4)	180 (63.6)
1	31 (33.3)	31 (32.3)	41 (43.6)	103 (36.4)

[a]Reported by investigator.
[b]Race is not allowed to be recorded in France.
[c]ECOG baseline data were missing for 2 patients in the placebo group (both patients had ECOG status = 0 at cycle 1).
ECOG performance status: 0 = fully active, able to carry on all predisease performance without restriction; 1 = restricted in physically strenuous activity but ambulatory and able to carry out work of a light or sedentary nature (eg, light housework, office work).
BMI = body mass index; CaM = calmangafodipir; ECOG = Eastern Cooperative Oncology Group; SAF = safety analysis set.

Supplementary Table 5. POLAR: analysis of moderate-to-severe CIPN at month 9 in Asian/non-Asian subgroups (mITT)

Patient group	n	Estimated event rate[a] (95% CI)	Relative risk[b] (95% CI)	*P* value
POLAR-A				
Asian				
CaM 5 µmol/kg	38	0.522 (0.333 to 0.818)	1.3417 (0.6818 to 2.6403)	.3948
Placebo	37	0.389 (0.234 to 0.645)	–	–
Non-Asian				
CaM 5 µmol/kg	82	0.602 (0.455 to 0.797)	1.6060 (1.0242 to 2.5182)	.0390
Placebo	82	0.375 (0.264 to 0.533)	–	–
POLAR-M				
Asian				
CaM 5 µmol/kg	29	0.555 (0.340 to 0.906)	1.3044 (0.6171 to 2.7572)	.4865
Placebo	28	0.426 (0.242 to 0.750)	–	–
Non-Asian				
CaM 5 µmol/kg	26	0.411 (0.228 to 0.743)	0.8943 (0.4007 to 1.9962)	.7851
Placebo	29	0.460 (0.267 to 0.792)	–	–

Based on Cochran–Mantel–Haenszel analysis adjusted for cumulative dose of oxaliplatin.
[a]Estimates of event rates per treatment arm of moderate-to-severe CIPN according to the first 4 items of the Functional Assessment of Cancer Therapy/Gynecologic Oncology Group-Neurotoxicity-13-item subscale, targeting numbness, tingling, or discomfort in hands and/or feet (FACT/GOG NTX-4) 9 months after the first dose of study treatment for the observed mean cumulative dose of oxaliplatin (mg/m^2).
[b]Relative risk = ratio of the of the estimated event rates of CaM 5 µmol/kg vs placebo.
CaM = calmangafodipir; CI = confidence interval; CIPN = chemotherapy-induced peripheral neuropathy; mITT, modified intention-to-treat.

Supplementary Table 6. Study treatment and oxaliplatin exposure (mITT)

	POLAR-A		POLAR-M		
	CaM 5 µmol/kg (n = 138)	Placebo (n = 140)	CaM 5 µmol/kg (n = 78)	CaM 2 µmol/kg (n = 80)	Placebo (n = 78)
Patients completing 12 cycles of study treatment, n (%)	50 (36.2)	57 (40.7)	35 (44.9)	34 (42.5)	28 (35.9)
Patients completing 12 cycles of oxaliplatin, n (%)	39 (28.3)	61 (43.6)	37 (47.4)	36 (45.0)	27 (34.6)
Oxaliplatin dose intensity, mean (SD), mg/m^2 per 2 weeks	72.70 (10.30)	70.80 (12.00)	71.60 (12.30)	70.90 (11.20)	72.90 (11.80)
Cumulative dose of oxaliplatin 9 months after first dose of study drug, LSM (95% CI), mg/m^2	747.3 (705.5 to 789.2)	755.0 (713.3 to 796.6)	803.5 (734.0 to 873.1)	780.7 (711.9 to 849.5)	764.5 (694.9 to 834.1)

CaM = calmangafodipir; CI, confidence interval; mITT = modified intention-to-treat; LSM = least-squares mean; SD = standard deviation.

Supplementary Table 7. POLAR: change from baseline in time to complete the grooved pegboard test with the nondominant hand at 9 months (combined mITT)

Treatment	n	Mean (range) time to complete at baseline, seconds	Change from baseline[a] (95% CI), seconds	Treatment difference[b] (95% CI), seconds	*P* value
CaM 5 µmol/kg	151	100.33 (30–268)	15.54 (8.17 to 22.91)	10.15 (0.20 to 20.10)	.0456
Placebo	157	107.48 (59–300)	5.39 (−1.85 to 12.62)	–	–

Based on analysis of covariance adjusted for baseline grooved pegboard time, study, and region (Asia/non-Asia).
[a]Estimates of least-squares means of change from baseline in time to complete the grooved pegboard.
[b]Treatment difference = CaM 5 µmol/kg vs placebo in least-squares means of change from baseline.
CaM = calmangafodipir; CI = confidence interval.

Supplementary Table 8. POLAR-A: hypersensitivity SAEs

Patient	Preferred term	System organ class	Time to onset[a]	Severity	Relationship to study treatment	Action taken
CaM 5 μmol/kg						
A1	Infusion-related reaction	Immune system disorders	71	Moderate	Probably related	Dose not changed
A1	Infusion-related reaction	Immune system disorders	85	Severe	Probably related	Drug withdrawn
A2	Infusion-related reaction	Immune system disorders	71	Mild	Possibly related	Dose not changed
A2	Infusion-related reaction	Immune system disorders	113	Moderate	Definitely related	Drug withdrawn
A3	Anaphylactic reaction	Immune system disorders	69	Moderate	Probably related	Drug withdrawn
A4	Drug hypersensitivity	Immune system disorders	155	Life-threatening	Definitely related	Drug withdrawn
A5	Infusion-related reaction	Immune system disorders	119	Life-threatening	Definitely related	Drug withdrawn
A6	Anaphylactic reaction	Immune system disorders	107	Severe	Unlikely related	Dose not changed
Placebo						
A7	Pneumonitis	Respiratory, thoracic and mediastinal disorders	172	Moderate	Possibly related	Not applicable

Table presents SAEs from Hypersensitivity Standardised Medical Dictionary for Regulatory Activities Queries.
[a]Days from first dose of study treatment.
SAE, serious adverse event.

Supplementary Table 9. POLAR-M: hypersensitivity SAEs

Patient	Preferred term	System organ class	Time to onset[a]	Severity	Relationship to study treatment	Action taken
CaM 5 µmol/kg						
M1	Hypersensitivity	Immune system disorders	144	Mild	Not related	Dose not changed
M2	Anaphylactic shock	Immune system disorders	86	Severe	Not related	Dose not changed
M3	Drug hypersensitivity	Immune system disorders	197	Severe	Not related	Drug withdrawn
CaM 2 µmol/kg						
M4	Pneumonitis	Respiratory, thoracic, and mediastinal disorders	217	Severe	Unlikely related	Dose delayed
M5	Infusion-related reaction	Immune system disorders	1	Moderate	Probably related	Drug interrupted
M6	Urticaria	Immune system disorders	66	Mild	Not related	Dose not changed
Placebo						
M7	Urticaria	Immune system disorders	71	Mild	Not related	Dose not changed

Table presents SAEs from Hypersensitivity Standardised Medical Dictionary for Regulatory Activities Queries.
[a]Days from first dose of study treatment.
SAE = serious adverse event.

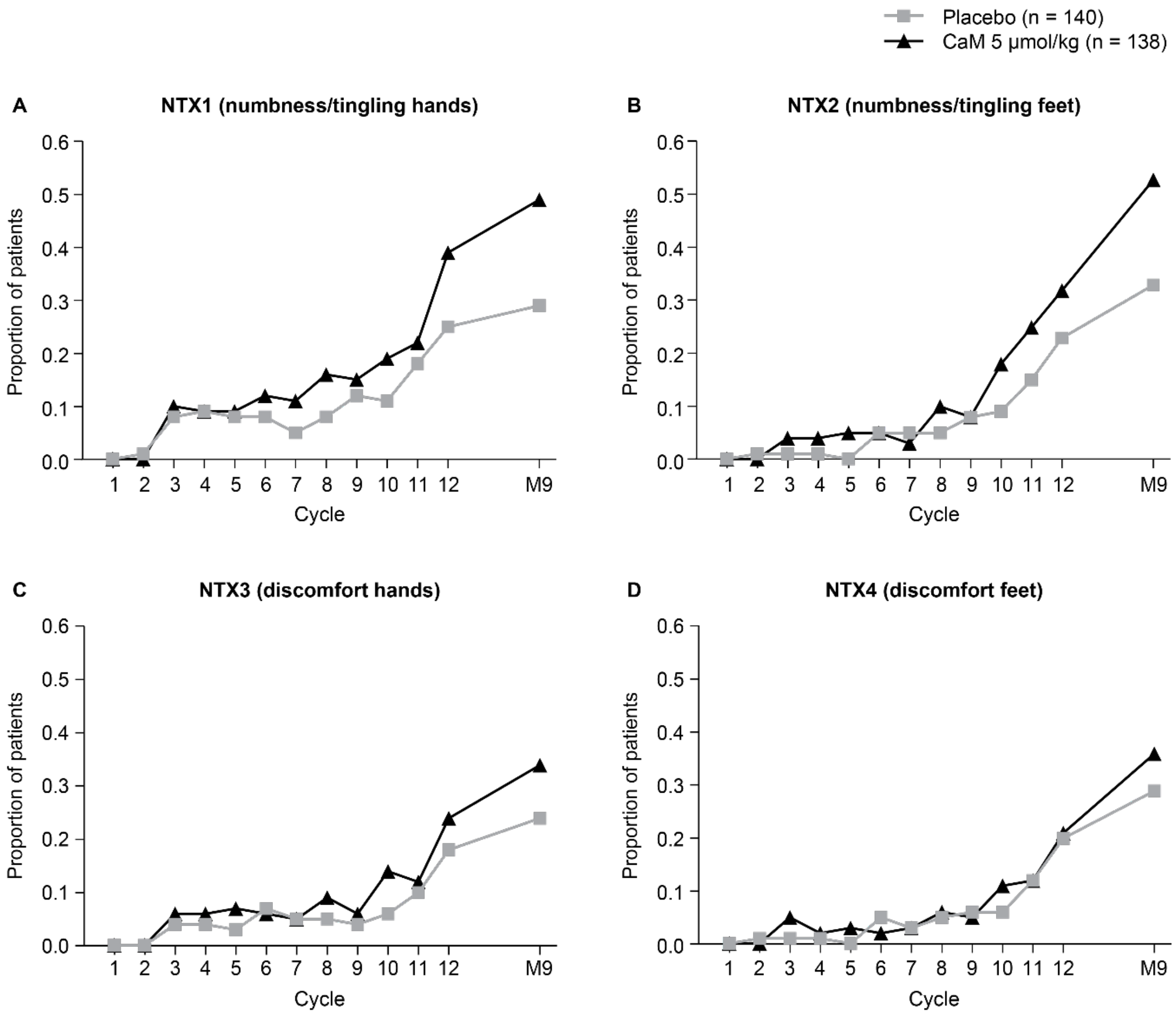

Supplementary Figure 1. POLAR-A: FACT/GOG-NTX-4 subscale items (mITT).

Proportions of patients at each treatment visit (cycle) and at 9 months (primary endpoint). The *y*-axis represents the proportion of patients reporting a score of 3 or 4 on the first (A), second (B), third (C), and fourth (D) items of the Assessment of Cancer Therapy/Gynecologic Oncology Group-Neurotoxicity-13-item subscale (FACT/GOG-NTX-13). CIPN = chemotherapy-induced peripheral neuropathy; M = month; mITT = modified intention-to-treat.

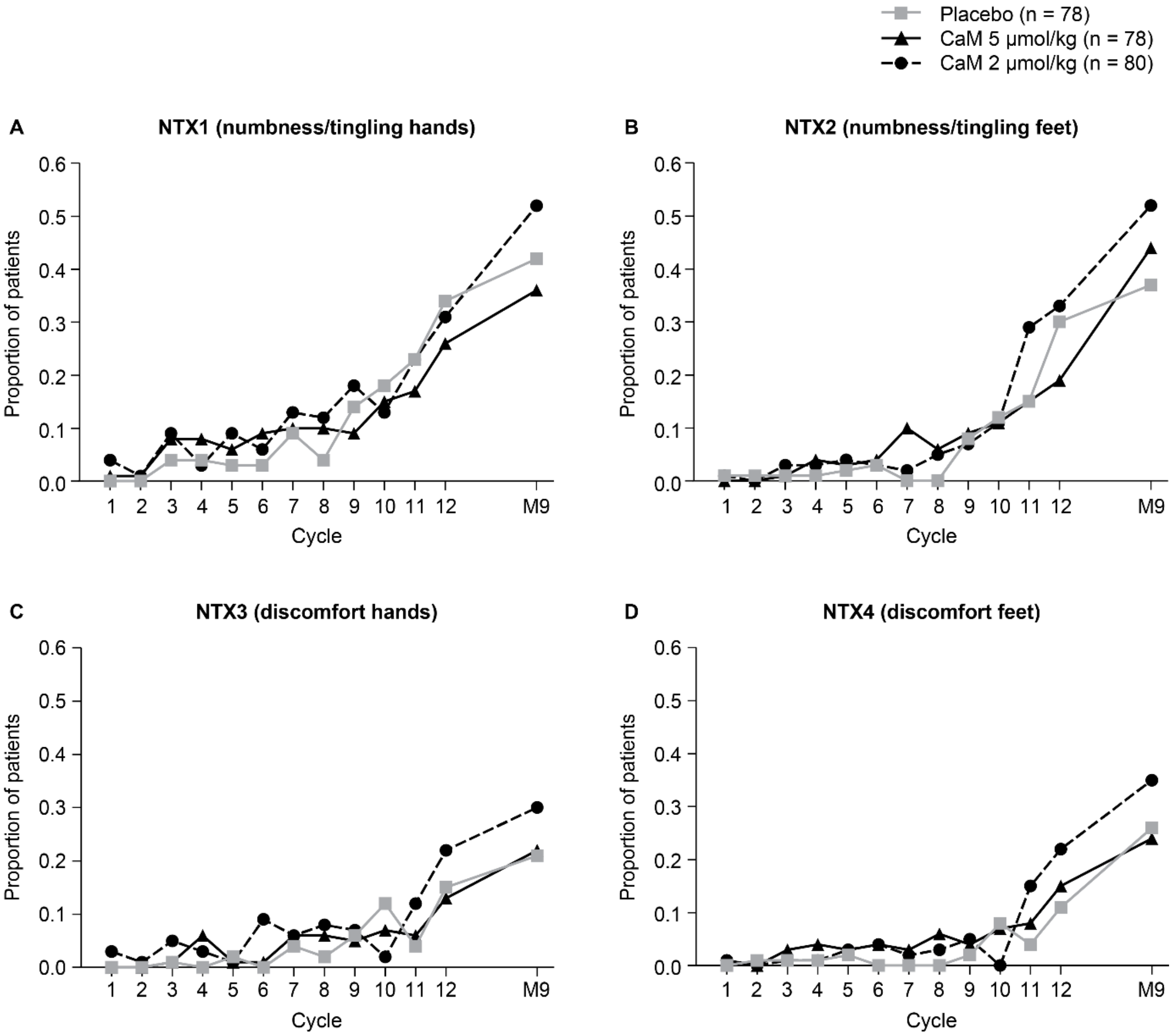

Supplementary Figure 2. POLAR-M: FACT/GOG-NTX-4 subscale items (mITT).

Proportions of patients at each treatment visit (cycle) and at 9 months (primary endpoint). The *y*-axis represents the proportion of patients reporting a score of 3 or 4 on the first (A), second (B), third (C), and fourth (D) items of the FACT/GOG-NTX-13. CIPN = chemotherapy-induced peripheral neuropathy;

FACT/GOG-NTX-13 = Assessment of Cancer Therapy/Gynecologic Oncology Group-Neurotoxicity-13-item subscale; M = month; mITT = modified intention-to-treat.

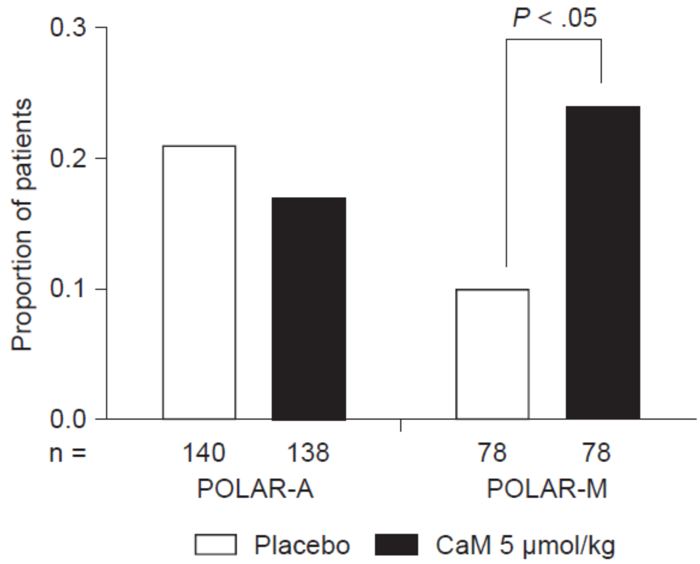

Supplementary Figure 3. Proportion of patients completing 12 cycles of both oxaliplatin and study treatment (mITT).

CaM = calmangafodipir; mITT = modified intention-to-treat.

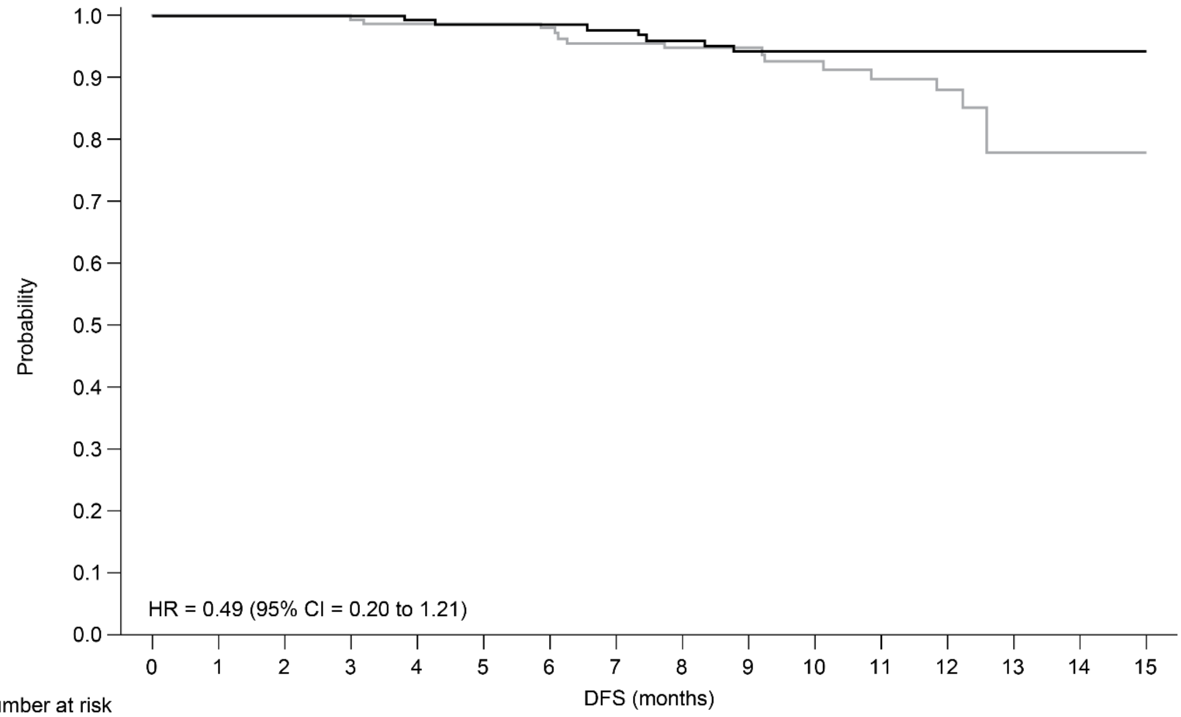

Number at risk

Placebo	150	143	143	141	137	137	130	122	114	100	69	63	47	9	8	6
CaM 5 µmol/kg	147	139	139	134	129	126	122	118	114	89	66	63	47	8	3	3

Placebo   CaM 5 µmol/kg

Supplementary Figure 4. POLAR-A: Kaplan–Meier plot of disease-free survival (SAF).

A DFS event was defined as signs or symptoms of recurrence of colorectal cancer or death of any cause. CaM = calmangafodipir; CI = confidence interval; DFS = disease-free survival; HR = hazard ratio; SAF = safety analysis set.

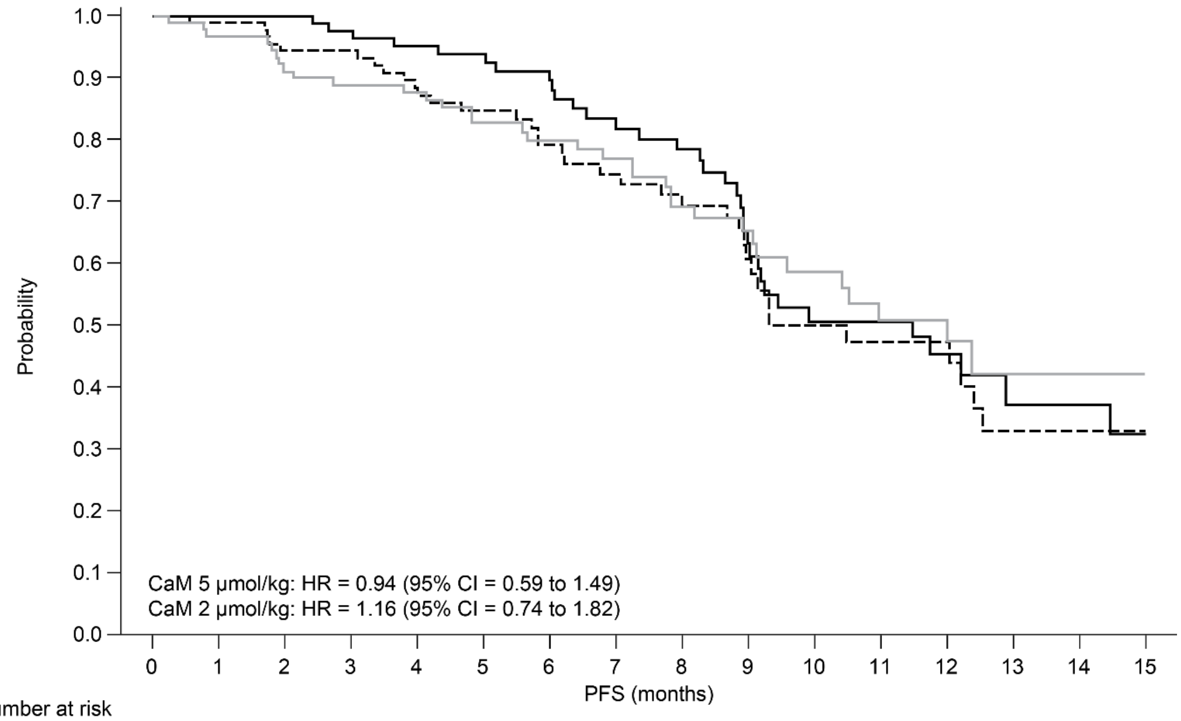

Number at risk

	0	1	2	3	4	5	6	7	8	9	10	11	12	13	14	15
Placebo	95	89	79	76	73	67	56	52	41	32	23	20	15	7	7	7
CaM 5 μmol/kg	93	88	84	80	75	70	60	51	47	33	23	22	16	8	8	6
CaM 2 μmol/kg	95	90	83	81	72	67	54	46	41	26	18	16	14	9	9	6

Supplementary Figure 5. POLAR-M: Kaplan–Meier plot of progression-free survival (SAF).

A PFS event was defined as progressive disease or death of any cause. Two patients were excluded from the analysis (incorrectly enrolled in the study). CaM = calmangafodipir; CI = confidence interval; HR = hazard ratio; PFS = progression-free survival; SAF = safety analysis set.

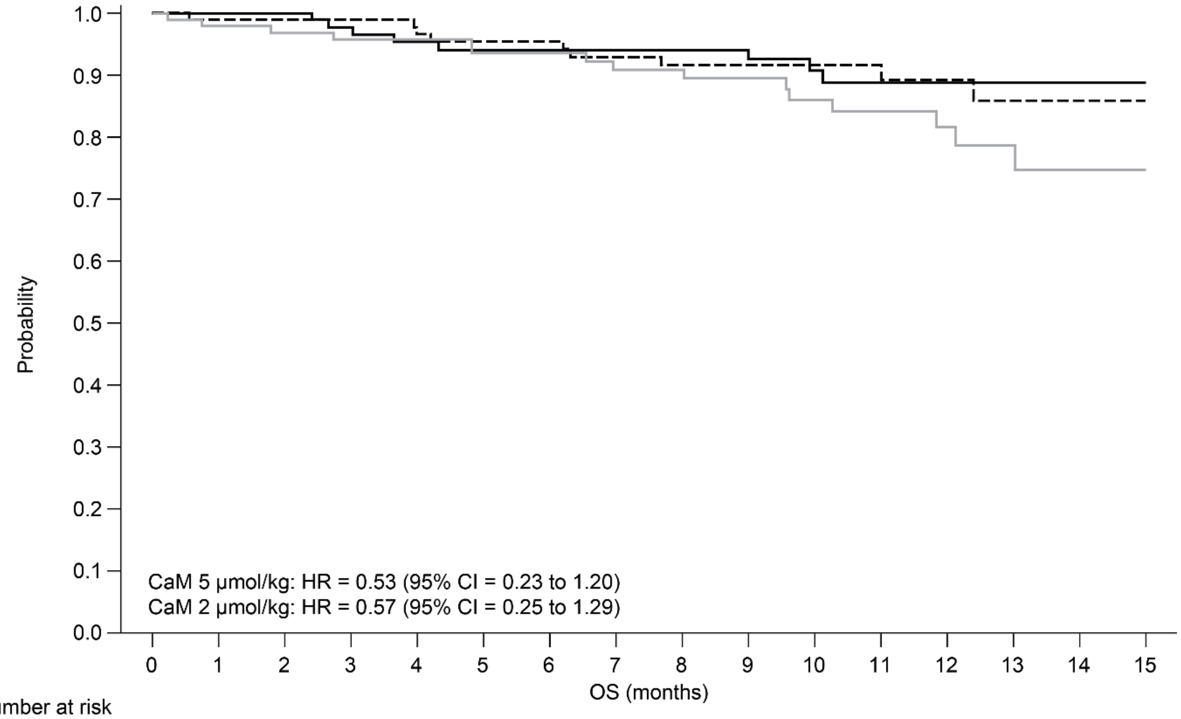

Number at risk																
Placebo	95	92	89	86	85	83	77	70	67	58	47	43	30	19	14	10
CaM 5 μmol/kg	93	91	89	85	80	78	75	69	65	62	47	44	38	25	23	16
CaM 2 μmol/kg	95	91	90	89	85	82	77	72	67	56	44	41	35	24	22	15

Placebo — CaM 5 μmol/kg — CaM 2 μmol/kg

Supplementary Figure 6. POLAR-M: Kaplan–Meier plot of overall survival (SAF).

An OS event was defined as death of any cause. Two patients were excluded from the analysis (incorrectly enrolled in the study). CaM = calmangafodipir; CI = confidence interval; HR = hazard ratio; OS = overall survival; SAF = safety analysis set.
